# Complication of endoscopic vacuum therapy in anastomotic leak: closure of iatrogenic small bowel fistula using a novel helix tacking device

**DOI:** 10.1055/a-2638-5325

**Published:** 2025-07-17

**Authors:** Ahmed Alwali, Clemens Schafmayer, Imad Kamaleddine

**Affiliations:** 1 39071 Department of General, Visceral, Thoracic, Vascular and Transplant Surgery, Rostock University Medical Center, Rostock, Germany

Endoscopic vacuum therapy is an effective technique for managing colorectal anastomotic leaks 1 . However, its use in cases of intra-abdominal anastomotic leaks should be performed with caution in experienced centers due to the high risk of complications, such as iatrogenic fistulas 2 . This case highlights a novel endoscopic suturing approach to close a small bowel fistula that developed as a complication of endoscopic vacuum therapy for an anastomotic leak after colorectal surgery.

A 66-year-old man underwent Hartmann’s reversal 6 months after surgery for perforated sigmoid diverticulitis. One week postdischarge, he presented with abdominal pain and elevated infection markers. A computed tomography scan and endoscopy confirmed an anastomotic leak 16 cm from the anal verge with an extra-luminal cavity. As the patient showed no signs of peritonitis and the 18th postoperative day was not ideal for reoperation or stoma diversion, conservative management was chosen. After endoscopic lavage, endoscopic intraluminal vacuum therapy (EndoSponge; B. Braun, Melsungen, Germany) was initiated, along with antibiotics therapy and a liquid diet.

Over 10 days, the patient’s condition improved. However, he developed new abdominal pain with rising infection markers. Small bowel contents were observed draining from the lower pole of the laparotomy wound. Endoscopy revealed an opening in a small bowel loop within the insufficiency cavity, suggesting a fistula likely caused by vacuum therapy ( Fig. 1 ).

**Fig. 1:**
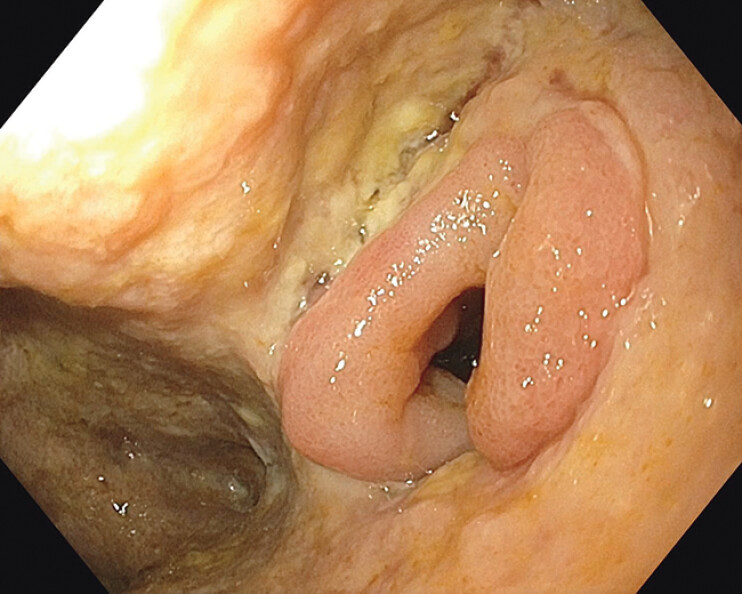
Endoscopic view of an opening in a small bowel loop within the insufficiency cavity, suggesting a fistula likely caused by vacuum therapy.

Initial attempts to close the fistula with endoscopic clips failed. The daily fistula output was 1,500 mL. Endoscopic suturing with a novel helix tacking device (X-Tack, Apollo Endosurgery, Austin, TX) was performed, reducing output significantly to 100 mL/d ( Fig. 2 ). Vacuum therapy continued for 2 weeks with sponge changes every 3 days to promote granulation. The output decreased to 20 mL/d and oral intake was gradually resumed. The patient was discharged in good condition for outpatient follow-up. Three-month follow-up showed a chronic cavity with granulation tissue but no persistent fistula. No discharge was observed from the laparotomy wound ( Video 1 ).

**Fig. 2:**
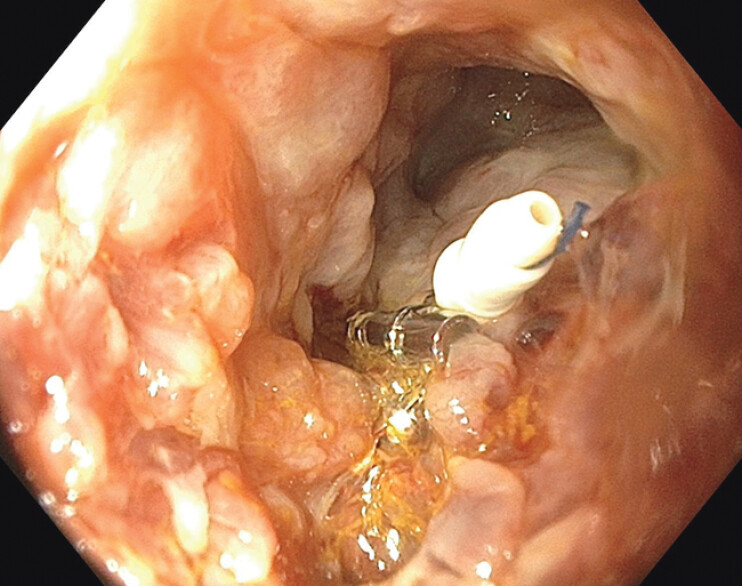
Closure of small bowel fistula using the X-Tack device.

This video demonstrates a novel approach for closing an iatrogenic small bowel fistula within the anastomotic insufficiency cavity after colorectal surgery using the X-Tack device.Video 1

Endoscopy_UCTN_Code_CPL_1AJ_2AJ

## Notes

Endoscopy E-Videos https://eref.thieme.de/e-videos
*E-Videos* is an open access online section of the journal *Endoscopy* , reporting on interesting cases and new techniques in gastroenterological endoscopy. All papers include a high-quality video and are published with a Creative Commons CC-BY license. Endoscopy E-Videos qualify for HINARI discounts and waivers and eligibility is automatically checked during the submission process. We grant 100% waivers to articles whose corresponding authors are based in Group A countries and 50% waivers to those who are based in Group B countries as classified by Research4Life (see: https://www.research4life.org/access/eligibility/ ). This section has its own submission website at https://mc.manuscriptcentral.com/e-videos .
